# The Activity Rhythm and Home Range Characteristics of Released Chinese Pangolins (*Manis pentadactyla*)

**DOI:** 10.3390/ani15111658

**Published:** 2025-06-04

**Authors:** Haochen Huang, Zhenhui Shen, Xinhao Zhang, Hongyi Cheng, Chungang Xie, Rongquan Zheng

**Affiliations:** 1 College of Life Sciences, Zhejiang Normal University, Jinhua 321004, China; 19852999776@163.com (H.H.); 19550597244@163.com (Z.S.); star_233@163.com (X.Z.); chy1944@zjnu.cn (H.C.); 2 Jinhua Wildlife Conservation and Management Station, Jinhua 321000, China; zhelin311300@126.com; 3 Xingzhi College, Zhejiang Normal University, Jinhua 321004, China

**Keywords:** *Manis pentadactyla*, accelerometer analysis, habitat utilization, GPS technology, reintroduction

## Abstract

As a mammal with unique ecological habits, the Chinese pangolin, *Manis pentadactyla,* exhibits nocturnal behavior and has a hidden, cave-dwelling lifestyle. Severe habitat degradation and fragmentation have led to a rapid decline in its wild population. Moreover, the immature state of artificial breeding technology restricts the availability of research samples, particularly with regard to behavioral and ecological data. This makes it extremely difficult to continuously monitor the pangolin’s ecological habits and population dynamics for a long time. The comprehensive research archive is yet to be established, greatly limiting the in-depth understanding of this species’ ecological behavior. To mitigate this data shortage, this study installed GPS positioning and accelerometer technology devices on five Chinese pangolins that were rescued from Zhejiang and released into the wild. Activity rhythms and home ranges were then analyzed based on the collected data. The results showed that all the five released Chinese pangolins were active in the wild and survived throughout the tracking period (21–72 days). The pangolins were mainly active at night, with activity starting after 18:00 and being mainly concentrated between 22:00 and 4:00. The home range of the Chinese pangolins based on MCP was 777.41 ± 426.26 hm^2^, and the core range based on KDE was 596.21 ± 265.58 hm^2^ (50% KDE). This result confirms that high-frequency GPS positioning combined with accelerometer data can effectively determine the home range sizes, habitat utilization, and fine-scale activity rhythms of wild-released pangolins.

## Simple Summary

GPS positioning technology has become an effective means for researchers to study wildlife and protect the biodiversity of the Earth. It can obtain the spatial location, movement, and time information of wildlife and is widely used in the field of wildlife conservation. The Chinese pangolin (*Manis pentadactyla*) has become an endangered flagship species due to its unique morphological characteristics (e.g., scale-covered body), specialized ant-/termite-eating behavior, and critical conservation status. As a charismatic yet cryptic species, it plays a key role in mobilizing public support for biodiversity conservation efforts. This study used GPS positioning and accelerometer technology to analyze the activity rhythm and home range of five Chinese pangolins released into the wild. The results showed that the nighttime activity of the pangolins began after 18:00, mainly concentrated between 22:00 and 4:00, and the core range of activity was 596.21 ± 265.58 hm^2^ (hectares). These data are necessary for related areas such as evaluating *Manis pentadactyla* habitat quality, habitat selection, and species carrying capacity evaluation. This study highlights how advanced tools like accelerometers can enhance the understanding of cryptic behaviors, aiding targeted conservation efforts for this endangered species.

## 1. Introduction

The Chinese pangolin (*Manis pentadactyla*; Gray, 1827) is a placental mammal covered with overlapping scales with a unique diet, preying on ants and termites [1,2]. This species is distributed in Southeast Asian countries such as China, Nepal, and Myanmar. It is similar to other tree-dwelling pangolin species and can climb trees and swim. Most of the time they live in underground burrows [3]. *Manis pentadactyla* is solitary and rarely lives with other individuals except during mating or offspring rearing [4]. The mating season is concentrated in summer and autumn, during which male pangolins usually leave odors and traces near their burrows to lure females [5]. As a timid mammal that lurks during the day and goes out at night, it is highly secretive and is difficult to study in the wild. Thus, its behavior and habits still have many mysteries.

Wild release is a strategy of reintroducing to the wild animals that have been artificially bred or rescued from the wild, to increase the population size [6,7]. For the critically endangered National First-class Protected Animal Chinese pangolin, habitat shrinkage and fragmentation are second only to human interference in causing a decline in its wild population. In conservation biology, in situ or ex situ release is important for maintaining the long-term survival of wild populations [8]. Wildlife released into the wild tend to explore more after leaving captivity. Then, as they become increasingly familiar with environmental resources, their utilization patterns and activity rhythms will change [9]. Therefore, in order to effectively protect this species, the most targeted and effective measure is to implement a scientific wild-release plan for captive individuals and simultaneously carry out comprehensive and accurate tracking work. In a *Panthera tigris altaica* conservation project, GPS collar tracking provided data on migration routes and territories, enabling the creation of ecological corridors and protected areas. This led to a 20% increase in suitable habitats over 5 years [10]. For African elephants, combined GPS and accelerometer data illuminated daily activities and behaviors, reducing human–elephant conflicts and safeguarding both the elephants and the local communities [11]. By utilizing advanced GPS and accelerometer technology, the activity trajectory, habitat utilization, and activity rhythm of released pangolins can be continuously monitored and analyzed. In this study, accelerometers were used to measure movement intensity (e.g., steps and three-axis acceleration), providing fine-scale data on daily activity patterns. Based on this, protection strategies can be dynamically adjusted to ensure that the release action can effectively improve the wild population size and quality of life of Chinese pangolins.

GPS technology is an effective means of locating released wildlife and analyzing their habitat selection. Compared with traditional visual observation methods and eDNA technology [12], GPS tracking has lower cost, smaller size, and less impact on pangolin behavior. This advancement enables more-precise localization of wild pangolins. Additionally, the GPS tracking device features an extended battery life, capable of continuous operation for over six months. This significantly minimizes the interference to pangolins during equipment replacement [13]. In recent years, with the widespread application of satellite positioning and radio telemetry technology in wildlife tracking, home range research has become one of the hot topics in the conservation biology of previously neglected wildlife such as giant pandas (*Ailuropoda melanoleuca*), red pandas (*Ailurus fulgens*), lions (*Panthera leo*), tigers (*Panthera tigris*), etc. [14,15,16,17]. Although there have been many studies on the habitat utilization of other large mammals, there are almost no studies on the long-term monitoring of Chinese pangolins in the wild. Sun first summarized the technique of installing a VHF radio transmitter on the tail scale of the Chinese pangolin and recorded the cave use pattern and activity site of a female individual in Taiwan, China, using the radio telemetry method. However, the location time obtained was not continuous, and animals in motion were often not detected, leading to the interruption of the study [18]. Different from the Taiwanese pangolin (*M. pentadactyla pentadactyla*), the Chinese pangolin subspecies (*M. pentadactyla auritus*) has no stable population at present, so there is a lack of experimental individuals and cases that can be released on a large scale, with only one long-term tracked female case in Guangdong, China [18,19,20]. Importantly, studying the habitat and activity rhythm of wild-released pangolins can help identify the factors influencing individual spatial exploration and adaptability and provide a basis for the management of released populations. Extended monitoring can elucidate whether the welfare or survival potential of individuals has been improved through the rehabilitation process [21]. To study the activity rhythm of animals, methods of study using camera traps and voice recorders are often used [22,23]. However, new methods deliver the greatest effect. Corresponding to the GPS exploration of home range and habitat extent, accelerometers can be used to measure the pangolins’ movement volume, thereby indirectly reflecting their activity rhythms. They can monitor the acceleration changes of Chinese pangolins in three-dimensional space and convert these changes into digital signals for recording. In this study, the accelerometers were primarily used to measure parameters such as the movement volume of pangolins (expressed in units of gravitational acceleration, G) and the number of activity steps. Previous research has demonstrated that accelerometers can acquire authentic activity data on animals in their natural habitats [24]. This makes accelerometers a valuable tool for delving into animal behavior patterns and ecological habits. Releasing pangolins without monitoring via GPS and accelerometer technology, along with a lack of experimental data, may result in unclear survival situations and uncertainty about the adaptability to the wild environment of the released individuals, making it difficult to evaluate the effectiveness of the release activities. Given this, in order to explore the home range and activity rhythm of Chinese pangolins, this study used precise GPS positioning and accelerometer technology devices to track released Chinese pangolins that had been sheltered or rescued. This will provide solid data support and literary reference for the systematic conservation of wild-released pangolins. The subsequent development of scientific and reasonable conservation strategies will aid the long-term survival of this endangered species in nature.

## 2. Materials and Methods

### 2.1. Overview of the Release Location and the Five Pangolins

Starting from 14 June 2023, five Chinese pangolins were released into the wild, in their original rescue areas (Table 1). Multiple reports have documented pangolins in these regions, where the release sites are low-altitude pine forests or deciduous forests on mountain slopes—ideal hiding places for the species. The terrain has relatively small undulations, with slopes ranging from 20 to 40 degrees. There are many fallen trees and traces of termite activity. The dead leaf layer is thick, and the soil is loose, which is conducive to pangolins digging caves and moving between different areas. These areas are close to water sources, have abundant food, and are suitable for the wild survival of pangolins [4].

Before release, the morphological parameters of each pangolin were measured, including their body weight, body length, and tail length (Table 2).

### 2.2. Installation of Tracking Devices

The GPS positioning devices were custom-made by Hunan Global Messenger Technology Co., Ltd. (Changsha, China). Due to the nocturnal nature of the pangolins, the solar charging module was abandoned and replaced with a built-in lithium battery. The battery capacity is 1200 mAh, and the corresponding open circuit voltage is greater than or equal to 4.2 V when fully charged. During the monitoring period, all the devices remained connected until their battery was depleted.

Before release, morphological parameters were measured for each pangolin and a GPS positioning transmitter was attached to a single scale near the tail base. To minimize scale breakage or fragmentation, larger (width > 25 mm) and thicker (thickness > 2 mm) tail-base scales were selected as far as possible. Two holes (diameter 2.5 mm) were drilled more than 5 mm apart in the non-vascularized part of the scales to secure the bolts. The weight of the transmitter was 41 g, which was less than 2% of the pangolin’s weight [25]. Additionally, each GPS transmitter was integrated with a three-axis accelerometer (sensitivity threshold: 0.15 G) to record movement dynamics in real time. The accelerometer measured acceleration changes in the X (left–right), Y (front–back), and Z (up–down) directions, converting these into activity counts (e.g., steps) and intensity metrics. Data were stored in the device’s memory and later synchronized with GPS data for comprehensive activity analysis. The ethical review was approved by the Experimental Animal Ethics Committee of Zhejiang Normal University.

**Figure 1:**
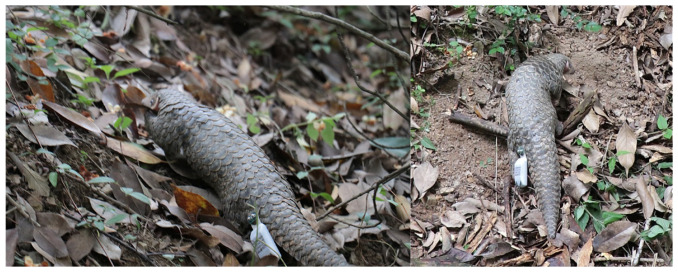
*M. pentadactyla* wearing GPS positioning devices (the individual on the left is MP04, and the one on the right is MP02).

The process of marking and installing GPS devices on released pangolins took only five minutes and was performed without the use of any anesthesia. After the installation of the device was completed, the pangolins were kept for 1–3 days in a wild-release training area in the Zhejiang pangolin protection and breeding base. There, they were allowed to adapt to the wild environment, including human presence, and were exposed to various simulated situations. Due to the cryptic nature and burrowing behavior of Chinese pangolins, device uninstallation and recovery post-monitoring were not feasible without causing additional stress to the animals. The GPS transmitters (41 g, <2% body weight) caused no observable behavioral impacts during the 1–3 days of pre-release acclimation, ensuring compliance with animal welfare standards for minimal disturbance [26] (Figure 1).

### 2.3. Collection of Release Data

#### 2.3.1. GPS Data

The satellite positioning technology of the tracking device was used to obtain information such as latitude, longitude, speed, heading, and altitude. Considering the power performance of the device, telemetry was conducted at 2 h intervals. Data were obtained through triangulation and direct GPS sampling, with transmission occurring after every five collected points. The equipment positioning accuracy was analyzed using linear regression: Error (meters) = 2.679243 × HDOP + 0.59144.

The data transmitted by GPS devices was used to track and analyze pangolin trajectories, record spatiotemporal positioning data at 2 h intervals, evaluate habitat utilization through 50% kernel density estimation (KDE) core activity zones, and determine daily activity rhythms by quantifying movement events (≥5 m displacement) across diurnal cycles. Specifically, nocturnal activity peaks were identified via temporal aggregation of the GPS-derived movement volume. These methods align with established practices in wildlife telemetry and have been validated for assessing mammalian behavioral ecology [27]. In this study, the movement volume was quantified as the total number of positional changes recorded by the GPS device during each 2 h data collection cycle. Specifically, a “movement event” was defined as a change in geographic coordinates exceeding a threshold of 5 m, based on the device’s positional accuracy (≤3 m; [15]). These events were aggregated per cycle to generate a measure of cumulative activity. Note: This term specifically refers to GPS-derived movement events and should not be confused with accelerometer-derived activity counts (see Section 2.3.2 for details).

The information on the five wild-released *M. pentadactyla* individuals is shown in Table 3. Based on continuous GPS transmissions and accelerometer activity counts (≥0.15 G threshold) throughout the 21–72-day monitoring period, no mortality signals (e.g., static acceleration for >24 h) were detected, and all the individuals exhibited stable diurnal activity rhythms. Although the devices were not recovered, post-release survival was inferred from consistent data streams and the absence of mortality-related patterns in the movement metrics.

#### 2.3.2. Accelerometer Data

The detection of accelerometer indicators is achieved through a three-axis acceleration sensor with built-in walking detection and step counting functions. Algorithmic processing of acceleration signals converts them into activity counts. The sensor measures real-time acceleration in the X (left–right), Y (front–back), and Z (up–down) directions, with a sensitivity detection threshold set to a default of 0.15 G. In static situations, such as during rest or after death, the accelerometer signal only represents the gravity acting on the sensor. When animals move, the sensor output represents the gravitational acceleration and the inertial acceleration generated by the motion [28]. Accelerometers include microprocessors and digital memory to store recorded measurement values until the device is retrieved [29]. When the tracker detects acceleration values in the X, Y, and Z directions exceeding the set threshold of 0.15 G, the device records an increase of 1 step, indirectly reflecting the intensity of the pangolin movement [30]. The accelerometer data were processed to derive activity counts, defined as the number of steps exceeding a 0.15 G threshold. This metric quantifies fine-scale movement intensity and is reported separately from the GPS-derived movement volume. Accelerators can continuously and for a long time record the activity data of animals and can obtain the real activity situation of animals in the natural environment, avoiding the errors and limitations that may be caused by manual observation [31].

### 2.4. Data Analysis

Since the calculated habitat size is the average movement distance within a time period, and as pangolins may not be moving in a straight line, this value is only a relative estimate. Some individuals lack complete time records due to unforeseen circumstances, such as pangolins staying in hidden burrows for a long time and unable to be located effectively, resulting in a certain degree of deviation when calculating their activity rhythms [32]. After removing abnormal points with positioning accuracy errors of over 2000 m, the Kolmogorov–Smirnov test was used to check whether they followed a normal distribution. Then, the repeated measures statistical method was used to examine differences in the movement volume of the pangolins at different time periods. Repeated measures analysis was conducted using linear mixed-effects models (LMMs). All statistical analyses were performed in R (v4.3.0) using the lme4 package (v1.1-33) for mixed models. Restricted maximum likelihood (REML) estimation was used to optimize the variance component calculations, particularly suitable for small sample sizes [33]. The analysis of the movement volume results is expressed as mean ± standard error (mean ± SE), and the activity altitude ranges are expressed as mean ± standard deviation (mean ± SD).

Home ranges were calculated using the minimum convex polygon (MCP) method [34], while the core area was estimated using the 50% kernel density estimation (KDE) method [35]. Using the fixed core method, the optimal bandwidth value was calculated using the Href parameter [36]. Then, the getverticeshr function in the package rgeos of R software (v4.3.0) was employed to generate contour lines within the home range and to calculate the size of the core area based on the Animal Movement Analyst plugin in Arcview3.x. The maps were drawn in ArcGIS and Google Earth Maps. The variables and their related statistical methods are presented in Table 4.

## 3. Results

### 3.1. Home Range

**Figure 2:**
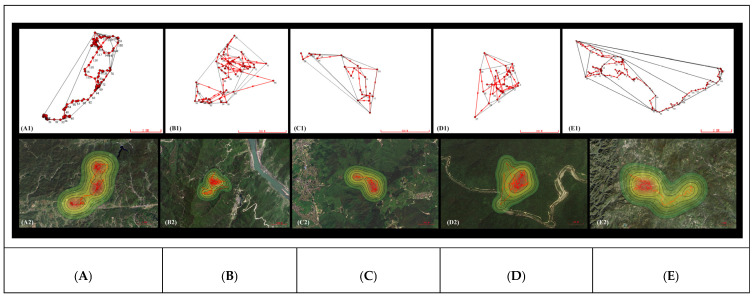
GPS point trajectories of five *M. pentadactyla* individuals; (**A1**–**E1**) are calculated by MCP model and (**A2**–**E2**) are by KDE model. The red area represents the core area. (**A**) MP01, (**B**) MP02, (**C**) MP03, (**D**) MP04, (**E**) MP05.

**Figure 3:**
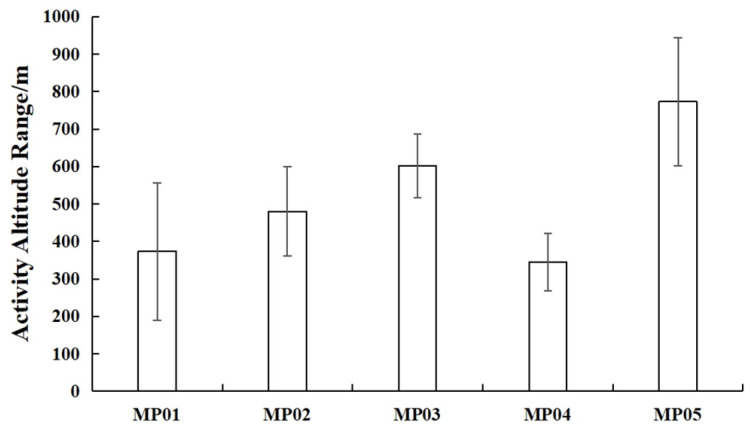
Activity altitude range of five *M. pentadactyla* individuals (Mean ± SD).

The home ranges of the five pangolins calculated based on the MCP were 777.41 ± 426.26 hm^2^, and the core area calculated based on KDE was 596.21 ± 265.58 hm^2^, reflecting significant inter-individual variation (Figure 2). The individual MP05 had the largest home range, with an overall activity area of 2027.39 hm^2^ and a core area of 1117.32 hm^2^, while MP04 had the smallest home range, with an overall activity area of 5.58 hm^2^ and a core area of 3.46 hm^2^ (Table 5). The KDE core area mirrored this pattern: MP05’s core area (1117.32 hm^2^) was 323 times larger than MP04’s (3.46 hm^2^; Table 5). The activity altitude range of the five wild pangolins is shown in Figure 3, with individual MP01 at 373.4 ± 183.39 m, MP02 at 480.11 ± 118.96 m, MP03 at 601.29 ± 84.44 m, MP04 at 344.8 ± 76.75 m, and MP05 at 773.1 ± 170.75 m.

### 3.2. Acceleration Activity Rhythm

The activity rhythm of the pangolins exhibited a clear nocturnal unimodal pattern, with peak movement intensity between 22:00 and 4:00 across all the individuals (Table 6). Notably, MP03 had the highest activity counts at 2:00 (3588 ± 161 steps), while MP05 showed sustained high activity from 22:00 to 2:00. All the individuals displayed significantly higher movement levels during nighttime hours than at dawn (6:00–8:00), with no significant differences at 20:00 (*p* > 0.05).

## 4. Discussion

### 4.1. Home Range Characteristics

The size and variation of the home range are important parameters for measuring the quality and carrying capacity of animal habitats, as well as for estimating the habitat area required to protect a minimum viable population of a species [37,38]. It can be used to measure the resource needs of wildlife and to provide important information for managing wildlife populations and their habitats. Home range formation and distribution are closely related to the animal’s sex, the season, the environment, etc. [39]. The core area (50% KDE) sometimes approached the size of the 99% MCP, a pattern that likely reflects the inclusion of infrequent outlier locations in the MCP calculations rather than strict behavioral fixation. For example, MP04’s MCP (5.58 hm^2^) and KDE (3.46 hm^2^) overlapped closely, which aligns with its release in a resource-dense low-altitude forest (Table 1), where limited movement was sufficient to meet foraging needs. Conversely, MP05’s larger MCP (2027.39 hm^2^) compared to its KDE (1117.32 hm^2^) suggests occasional long-distance movements, possibly influenced by mountainous terrain (Table 1) or longer captivity duration.

Although the MCP model is susceptible to the influence of specific loci, it can intuitively describe the overall characteristics of a home range. On the other hand, the KDE model can eliminate the influence of certain loci, making its description of the core area more accurate and supplementing the MCP calculation results [40]. The data from this study show that the size of the home range of *M. pentadactyla* varies significantly between different individuals. This variability can be attributed to differences in habitat quality (e.g., termite/ant abundance), release altitude, and individual physiological states (e.g., body mass, age). For example, MP05, released in spring (April 2024) with a shorter captivity period (3 months), exhibited the largest home range (2027.39 hm^2^ by MCP), likely influenced by seasonal termite activity at higher altitudes. In contrast, MP04, released in autumn (October 2023) after a longer captivity period (6 months), had the smallest home range (5.58 hm^2^), possibly due to reduced exploration behavior following extended acclimation to captive environments. These patterns may be related to seasonal environmental changes and human interference, as well as the length of captivity time. Springtime termite activity peaks have been documented in subtropical ecosystems [41], coinciding with post-hibernation foraging demands in mammals. In our study, MP05—released in spring and monitored for 72 days—exhibited the largest home range (2027.39 hm^2^; Table 4). While this suggests a potential link to seasonal resource dynamics, causal inference is constrained by the single spring-release replicate and variable monitoring durations across individuals. In late summer or early autumn, pangolins dig burrows in foraging areas to feed their offspring [4,42]. As a result, pangolins reduce their frequency and range of foraging in these seasons, leading to a decrease in their home range. MP05 (2.99 kg, 608 m release altitude, Table 1 and Table 2) had the largest MCP home range (2027.39 hm^2^), likely influenced by its high-altitude habitat, which features sparse vegetation and lower insect density. In contrast, MP04 (1.705 kg, 229 m release altitude) occupied the smallest home range (5.58 hm^2^), situated in a low-altitude area with dense termite mounds and abundant food resources. This relationship between body mass, release altitude, and home range size supports the resource distribution hypothesis, where larger individuals and high-altitude habitats require broader foraging areas to meet energy demands. Future habitat protection planning relies on this essential spatial scale information. By monitoring the correlation between the surrounding environment of habitat and pangolin activities during this period, the suitability of the habitat for pangolin survival can be accurately evaluated, providing scientific support for the optimization and management of the habitat.

The relatively small core area (50% KDE: 3.46–1117.32 hm^2^; Table 4) suggests strict dependence on localized resources, such as termite mounds and suitable burrowing soil (Table 1). Habitat destruction—including human-induced fragmentation or natural disturbances—could severely limit migration options for this slow-moving species, underscoring the urgency of protecting core activity zones (KDE 50% areas). The high-frequency activity traces within the core area indicate that this area has ideal digging conditions and abundant and stable food resources, making it the primary location for a pangolin to forage, survive, and reproduce. Occasional location records outside the core area suggest that the pangolin is tentatively searching for food sources in response to changes in its core area. Specifically, Chinese pangolins (*M. pentadactyla*) require low-to-mid-altitude forests with mixed deciduous and coniferous vegetation, which provide both burrowing substrates and insect prey [43]. This differs from tree-dwelling pangolin species like Manis javanica, which rely on arboreal habitats and have shorter burrowing needs [30]. Compared to African species (Smutsia spp.), *M. pentadactyla* exhibits stronger dependence on terrestrial burrows for thermoregulation and predator avoidance, likely due to its evolutionary adaptation to mountainous terrains in southern China [44]. Based on this, considering the feasibility of local law enforcement and the socio-economic impact, the core area of wild-released pangolins can be designated as an ecological red line area with no unauthorized entry. At the same time, ecological corridors and other protective measures can be established in the next step of research to simulate natural ecological connection channels and aid gene exchange, effectively preventing inbreeding as a result of habitat isolation. This will maintain the genetic vitality and adaptive evolutionary potential of the population, enabling it to grow.

### 4.2. Activity Rhythm Patterns and Ecological Significance

Wild *M. pentadactyla* were studied to analyze their activity rhythms, revealing novel high-resolution data on nocturnal behavior patterns, which align with prior captive observations but extend findings to wild populations by documenting continuous activity from 22:00 to 4:00 and seasonal variations in the peak activity timing [45]. The ants and termites that pangolins mainly prey on are highly active at night, and the quiet environment at night creates ideal conditions for foraging. The pangolins’ activity curve peaks from 22:00 to 4:00, which is consistent with the results of wild pangolin population studies using infrared camera monitoring technology [46]. If there are human interference sources, such as nighttime construction noise, strong light pollution, etc., in the pangolin reserve during this period, it may hinder its normal behavior, which in the long run will lead to damage to the ecological services of the habitat. For example, among previously rescued pangolins, individuals have fled to nearby villagers’ homes due to disturbances from nearby construction activities.

The thick dead leaf layer at release sites, a habitat feature associated with termite abundance in *Manis* spp. [3], may support efficient foraging within compact core areas (50% KDE: 3.46–1117.32 hm^2^; Table 5). When the food near the burrow decreases, the pangolin will migrate in search of additional sources of food and will dig new burrows for shelter. The pangolins mostly migrate in the early morning, frequently crossing ravines or water sources, presumably in search of food or shelter [43]. This can often result in a loss of telemetry data, as the tracker signal can be affected by mountains. During the observation period in this study, ineffective data on the pangolins were mostly received during this time.

In addition, the study also captured some activity trajectories during special periods, such as noon activity after rain. Post-rain activity deviations from the typical nocturnal pattern were occasionally observed (e.g., MP03 at 14:00 on Day 12; Figure 3), which aligns with experimental findings in *Manis* spp. [19,47]. These studies demonstrated that increased soil moisture improves ant nest accessibility, potentially explaining the observed daytime foraging events in our dataset. However, the triggers, seasonal variation, and individual differences of these special activity patterns are currently unknown. Meanwhile, further research is needed on the various behavioral types (such as resting, foraging, traveling) of pangolins. Long-term and larger-sample-sized monitoring projects are needed to identify the impact of environmental factors on the activity rhythm of pangolins. These studies should focus on using models and algorithms that are more suitable for field habitat selection.

### 4.3. Equipment Improvement

With the help of a new generation of high-precision GPS technology, the data from this study showed improved positioning accuracy in terms of data quality, effectively compensating for the inherent deficiencies in the spatiotemporal resolution of traditional methods such as direct observation and marker recapture. For example, human resources and visual constraints limit traditional observation, making continuous pangolin tracking difficult, whereas GPS data provides complete records of their activity, both day and night. Also, the marker-recapture method has long data intervals, missing some short-term behavioral changes in individuals, while GPS can provide real-time feedback.

However, the battery life and positioning capabilities of the device have long been a key issue that has plagued the ecological and conservation research of pangolins. The short positioning time and strong signal shielding were also technical challenges encountered in monitoring wild pangolins in this study, which inevitably led to errors in the data analysis process. Additionally, mountainous areas with complex terrain or dense vegetation interfere with satellite signals, resulting in periodic missing location data, which can have a significant impact on the compilation of a complete activity trajectory for pangolins and interfere with the analysis of their activity rhythms. According to previous research results, strong and flexible fastening methods and streamlined positioning devices are the main reasons for the extension of radio tracking cycles [48]. In the future, more advanced device labeling methods should be used to better fit the scales of pangolins to prevent them from falling off or being lost, while ensuring that they do not interfere with daily activities. At the same time, real-time, high-precision positioning should be increased to facilitate periodic recapture and equipment replacement in the wild, ensuring extended tracking times for pangolins. Very-high-frequency (VHF) or drone technology can be used to search and locate devices with depleted batteries, solving the problem of signal loss. While this study employed state-of-the-art tracking technology, future improvements may include adaptive fastening systems and hybrid energy solutions to address the challenges highlighted here.

## 5. Conclusions

The released Chinese pangolins (*M.pentadactyla*) exhibited distinct nocturnal activity rhythms as measured by the accelerometer data, with peak movement intensity occurring between 22:00 and 04:00, during which the greatest hours of activity occurred between 00:00 and 2:00. This is one of the adaptive characteristics of the Chinese pangolin, which is of vital importance for understanding its survival strategy. Home range sizes varied significantly among individuals, with an average of 777.41 ± 426.26 hm^2^ (MCP) and core activity areas of 596.21 ± 265.58 hm^2^ (50% KDE), influenced by factors such as release site resources and captivity duration. All the individuals successfully exhibited wild activity patterns, with no mortality recorded during monitoring, underscoring the viability of reintroduction efforts. The application of GPS and accelerometer technology proved effective in monitoring the released pangolins, providing valuable data on their home range and activity rhythm. Nevertheless, challenges like limited battery life and signal interference in complex terrains were encountered, suggesting that technological improvements are necessary for more efficient long-term monitoring. Although the study found that the released individuals exhibited stable activity rhythms during the monitoring period and no mortality signals were observed, the limited sample size and short tracking duration constrain the generalizability of these results. Therefore, we recommend exercising caution when applying the findings to broader conservation practices. Future research should involve larger sample sizes and longer monitoring periods to systematically assess the long-term survival and reproductive success of translocated individuals, thereby scientifically validating the feasibility and effectiveness of this strategy.

## Figures and Tables

**Table 1:** Information on the release locations of five *M. pentadactyla*.

Individual	Release Location	Coordinate	Release Altitude/m
MP01	Jinhua City, Zhejiang, China	E 11*.*3°, N 2*.*2°	191
MP02	Lishui City, Zhejiang, China	E 12*.*2°, N 2*.*8°	362
MP03	Yuyao City, Zhejiang, China	E 12*.*2°, N 2*.*8°	659
MP04	Lishui City, Zhejiang, China	E 11*.*6°, N 2*.*1°	229
MP05	Lishui City, Zhejiang, China	E 11*.*9°, N2*.*1°	608

* To prevent poaching of released *M. pentadactyla*, the specific latitude and longitude information of the release location is omitted from the table.

**Table 2:** Morphological parameters of five *M. pentadactyla*.

Individual	Sex	Weight/kg	Body Length/cm	Tail Length/cm
MP01	♂	2.09	58.0	21.5
MP02	♂	1.71	56.0	22.0
MP03	♂	2.85	58.0	22.0
MP04	♂	2.26	57.0	18.0
MP05	♂	2.99	64.0	24.0

**Table 3:** Monitoring information of five wild-released *M. pentadactyla*.

Individual	Release Time	Final Positioning Time	Monitoring Duration/d	Location Points
MP01	14 June 2023	8 August 2023	55	165
MP02	16 August 2023	10 September 2023	25	57
MP03	20 September 2023	9 November 2023	27	24
MP04	19 October 2023	6 November 2023	21	30
MP05	3 April 2024	14 June 2024	72	141

**Table 4:** Summary of variables and associated statistical methods used in the study.

Variable	Data Source	Description/Calculation Method	Statistical Method
Movement volume	GPS	Number of position changes exceeding 5 m in 2 h intervals	Linear mixed-effects model (LMM); REML estimation
Home range (total area)	GPS	99% Minimum convex polygon (MCP) calculated using all GPS positions	MCP method using “adehabitatHR” package in R
Core activity area	GPS	50% Kernel density estimation (KDE) using Href bandwidth	KDE method with “getverticeshr” in “rgeos” package
Activity altitude	GPS	Elevation data extracted from GPS records	Descriptive statistics (mean ± SD)
Activity counts	Accelerometer	Number of step events exceeding 0.15 G threshold in X/Y/Z axes	Repeated measures LMM; REML estimation
Peak activity period	Accelerometer	Aggregated step counts across 2 h time windows between 18:00 and 8:00	Tukey HSD post hoc test following LMM
Device-derived survival inference	GPS + Accelerometer	Continuous data stream without >24 h inactivity indicates survival	Observational inference based on sustained data transmission

**Table 5:** Home range characteristics of five pangolins based on MCP and KDE.

Model	Individual	Area/hm^2^	Perimeter/km
MCP (99%)	MP01	1576.8	18.33
	MP02	36.91	2.49
	MP03	240.36	10.35
	MP04	5.58	1.02
	MP05	2027.39	20.93
KDE (50%)	MP01	1270.26	21.36
	MP02	19.35	2.19
	MP03	57.07	10.84
	MP04	3.46	0.67
	MP05	1117.32	35.13

**Table 6:** Activity counts and statistical analysis of *M. pentadactyla* at different time periods from18:00 to 8:00 the next day (mean ± SE).

Time	Activity Counts				
	MP01	MP02	MP03	MP04	MP05
18:00	541 ± 507 ab	284 ± 105 c	0	199 ± 90 b	0
20:00	1334 ± 135 ab	1824 ± 337 ab	1326 ± 466 abc	1820 ± 338 ab	1524 ± 253 ab
22:00	2321 ± 163 a	2349 ± 380 a	2968 ± 612 ab	2263 ± 527 ab	2446 ± 227 ab
0:00	2301 ± 199 a	1798 ± 280 ab	2666 ± 726 ab	2953 ± 51 ab	2274 ± 225 ab
2:00	2338 ± 261 a	1952 ± 340 ab	3588 ± 161 a	3519 ± 168 a	2814 ± 238 a
4:00	2078 ± 350 a	1655 ± 255 ab	2896 ± 83 ab	2478 ± 48 ab	2150 ± 505 ab
6:00	43 ± 31 b	693 ± 144 bc	708 ± 238 bc	795 ± 283 ab	382 ± 98 b
8:00	17 ± 10 b	258 ± 118 c	279 ± 129 c	267 ± 172 b	169 ± 55 b

Different letters in the same column indicate significant differences in activity counts (HSD Tukey test; *p* < 0.05).

## Data Availability

The data generated from the study is clearly presented and discussed in the manuscript.

## Abbreviations

The following abbreviations are used in this manuscript:

RMS	Analysis of variance
MCP	Minimum convex polygon
KDE	Kernel density estimation
